# Paternal perinatal mental health and early child development: An outcome‐wide analysis

**DOI:** 10.1002/imhj.70104

**Published:** 2026-06-10

**Authors:** Honor Scarlett, Xavier Benarous, Angèle Consoli, Tina Montreuil, Sarah Lyon‐Caen, Claire Philippat, Judith van der Waerden

**Affiliations:** ^1^ INSERM Unit 1136, Institut Pierre Louis d'Epidémiologie et de Santé Publique (IPLESP) Sorbonne Université Paris France; ^2^ Department of Child and Adolescent Psychiatry Armand‐Trousseau Hospital (APHP) Sorbonne‐Université Paris France; ^3^ Department of Child and Adolescent Psychiatry Hôpital Universitaire de la Pitié‐Salpêtrière, Faculté de Médecine Sorbonne Université Paris France; ^4^ Centre de Référence des Maladies Rares à Expression Psychiatrique, Department of Child and Adolescent Psychiatry AP‐HP Sorbonne Université, Hôpital Universitaire de la Pitié‐Salpêtrière Paris France; ^5^ Department of Psychiatry McGill University Montréal Québec Canada; ^6^ Inserm U1209, CNRS UMR 5309, Team of Environmental Epidemiology applied to Development and Respiratory Health University Grenoble Alpes, Institute for Advanced Biosciences Grenoble France

**Keywords:** anxiety, child, depression, development, father, mental health, socioeconomic

## Abstract

Paternal mental health (PMH) has been shown to associate with child development as early as infancy. However, the moderating effects of certain contextual factors remain poorly understood. Using data from the French SEPAGES cohort, an outcome‐wide analytic approach was used to investigate the relationship between PMH during pregnancy (*n* = 166), 0–12 months postnatally (*n* = 117), and 12–24 months postnatally (*n* = 207) with child socioemotional, behavioral and cognitive outcomes at two and three years. Mother's mental health, child sex and parent's professional class were then tested as potential moderators. Findings revealed no significant associations between paternal depression or anxiety with any measure of child development. However, mother's professional class and maternal prenatal depression moderated the association between paternal anxiety and several areas of child development, with children of lower maternal professional classes showing poorer cognitive outcomes but improved social perception when exposed to paternal anxiety. Children exposed to paternal anxiety showed worse working memory outcomes and future‐oriented tasks and organization when also exposed to maternal depression. While our findings contrast with current research, showing no relationship between PMH and child development, they highlight the importance of considering family socioeconomic position when studying the relationship between PMH and child development.

## 1 INTRODUCTION

Influences during the first 1000 days of a child's life, starting from conception, are crucial in predicting the course of subsequent development. In particular, numerous aspects of child socioemotional, cognitive and behavioral development have been seen to depend on both pre‐ and postnatal parental mental health (Spry et al., 2020; Stein et al., 2014). Although less studied, accumulating evidence indicates that paternal mental health (PMH) may affect child development to an equal extent as maternal mental health (Kane & Garber, 2004; Van Batenburg‐Eddes et al., 2013; Velders et al., 2011). A grouped meta‐analysis saw PMH to have a significant impact on all studied domains of child development, with a roughly 50% higher risk for adverse developmental outcomes in children of fathers experiencing mental illness (Scarlett et al., 2023). These findings are important, as global research suggests that approximately 2%–51% of fathers are susceptible to anxiety in the perinatal period (Ballard & Davies, 1996; Philpott et al., 2019), and 5%–10% of fathers to perinatal depression (Paulson & Bazemore, 2010), with similar figures seen in France (Nakamura et al., 2020). Comorbidity between paternal anxiety and depression is also extremely common, with almost half (45.7%) of adults with lifetime major depressive disorder also presenting one or more lifetime anxiety disorders worldwide (Kessler et al., 2015).

However, current evidence investigating the relevance of fathers’ specific mental health diagnosis in relation to their child's development is mixed. One study saw only paternal depression, and not paternal stress nor anxiety, to associate with adverse child development (Jones et al., 2023), whilst a recent meta‐analysis (Spry et al., 2020) reported no difference in effect size when comparing the effect of paternal anxiety or depression on child outcomes. Theoretically, there is evidence to support different pathways by which each condition may affect child development; Paternal depression often leads to withdrawal (Cameron et al., 2016; Kim & Swain, 2007), which may result in reduced responsiveness and engagement amongst children (Cheung & Theule, 2019; Spry et al., 2020), whereas paternal anxiety more commonly associates with heightened control, intrusiveness, or emotional reactivity (Chhabra et al., 2020; Philpott et al., 2019), potentially impacting children's autonomy and emotional regulation (Spry et al., 2020). Considering the heterogeneity in prevalence (Ballard & Davies, 1996; Nakamura et al., 2020; Paulson & Bazemore, 2010; Philpott et al., 2019) and risk factors (Chhabra et al., 2020) between paternal anxiety and depression, investigation of both disorders is necessary.

The literature has also focused primarily on postnatal PMH. Pregnancy has been shown to be a demanding period for fathers’ psychological needs (Ballard & Davies, 1996; Philpott et al., 2017), with feelings of incompetence, insecurity and anxiety for their partner's wellbeing regularly cited across qualitative studies (Baldwin et al., 2019; Deave et al., 2008; Pilkington & Rominov, 2017). Inclusion of prenatal PMH is thus imperative in discourse surrounding father's mental health.

Evidence suggests the effect of PMH may vary based on the area of child development, with lower effect sizes seen for indicators of autism (Ayano et al., 2019) than for internalizing or externalizing behaviors (Scarlett et al., 2023). Specific outcomes associate with distinct developmental pathways and may thus be differentially impacted; For example, PMH may impact child cognition through changes to the quality and quantity of cognitive stimulation (Passareli‐Carrazzoni et al., 2018; Rollè et al., 2019), whereas socioemotional or autism‐related traits may be more strongly shaped by parent‐child bonding, external social interactions, or genetic factors (Ayano et al., 2019; Cui et al., 2020; Cicchetti, 2016). However, comorbidity amongst child developmental disorders remains common (Ayoub & Fischer, 2006). Outcome‐wide analysis, proposed by VanderWeele (2017), may be a potential method to address this. By allowing the joint modelling of multiple outcomes, a comprehensive view of the relationship between PMH and child development can be discerned whilst allowing correlations amongst multiple outcomes to be considered (Anguita‐Ruiz et al., 2023).

Both developmental psychology and social epidemiology frameworks draw on the premise that early childhood development is shaped by the immediate home environment as well as broader social and structural factors that may influence family mental health and child outcomes. Paternal and maternal mental illness are highly interdependent (Ballard & Davies, 1996; Darwin et al., 2021), with the co‐occurrence of mental illness in both parents significantly increasing the risk of major depression and anxiety in their children (Merikangas et al., 1988). Research also indicates that PMH can moderate the impact of maternal mental health on child development (Ballard & Davies, 1996; Connell & Goodman, 2002; Dietz et al., 2009; Mezulis et al., 2004). Where present, the psychological stability of one parent may provide emotionally consistent and responsive caregiving as a protective factor against the effects of another's distress (Nakamura et al., 2020; Spry et al., 2020). Conversely, if both parents experience poor mental health, the cumulative stress and reduced caregiving capacity may compound risks for the child's socioemotional and cognitive development. Whilst studies show the association between PMH and their children's subsequent development of behavioral and emotional problems to remain significant after controlling for maternal mental health (Ramchandani et al., 2005; 2011), few studies have explored the potential moderating effect of mother's mental health on the effect of PMH.

The interaction between PMH and child development may also be partially moderated by child gender or sex assigned at birth—henceforth referred to as child sex. However, the direction appears inconsistent, with some reviews showing greater effect sizes for boys (Cheung & Theule, 2019) or girls (Connell & Goodman, 2002), and others seeing no difference (Kane & Garber, 2004; Wickersham et al., 2020). Further research is important, as these associations have not been explored extensively for PMH, and gender differences have previously been highlighted in child socioemotional and behavioral developmental domains (Chaplin et al., 2005; Else‐Quest et al., 2006). Finally, research investigating the association between PMH and child development must consider the wider pre and postnatal environment. Both personal and structural factors are known to influence the risk of PMH, such as the co‐parent relationship (Nakamura et al., 2020; Philpott et al., 2022), father's education level (Ramchandani et al., 2008), and socioeconomic position (SEP) (Ballard & Davies, 1996). Moreover, several of these factors are also known to associate with the course of child development (Cano, 2022; Hanington et al., 2012; Davis‐Kean, 2005) and may therefore exacerbate, or buffer, the impact of PMH. For example, research indicates that lower paternal education levels predict externalizing behavioral problems at 2–3 years (Letourneau et al., 2019) and psychosocial problems and IQ from 5–8 years (Cave & Wright, 2022; Mieloo et al., 2012). However, key socioeconomic moderators are still yet to be determined.

**Key points**

- Neither pre‐ nor postnatal paternal mental health (PMH) associated with any area of child development at two and three years of age, but both maternal depression and mother's professional class moderated the association between PMH and certain child outcomes.
- These findings highlight the importance of parental socioeconomic position (SEP) and coparent mental health when considering the relationship between father's mental health and child development.
- Whilst more research is needed on this topic, public health policy and outreach relating to families of lower SEP should take into account the concurrent mental health of both parents to better support child outcomes.

**Relevance to infant and early childhood mental health**

The study found a non‐significant relationship between father's anxiety and depression across all studied child outcomes at both two and three years. However, mother's professional class was revealed to be a recurrent moderator on the relationship between paternal anxiety and several child outcomes. These findings are likely influenced by this sample's socioeconomic position, supporting the need for greater inclusion of less privileged families often underrepresented in research on father's mental health. The importance of paternal anxiety, as opposed to depression, also highlights its relevance to child outcomes and defends the increased inclusion of paternal anxiety in the discussion surrounding early child development.

This study aims to provide a comprehensive assessment of the relationship between PMH and multiple domains of early child development, encompassing socioemotional, cognitive, and behavioral domains, in an outcome‐wide analysis. Both paternal depression and anxiety will be considered respectively, as well as the timing of exposure. All associations will be subsequently explored in relation to the mother's mental health, child sex, and parental SEP.

## 2 METHODS

### 2.1 Population and study design

The study population was taken from the French SEPAGES couple‐child cohort. SEPAGES recruited pregnant women from eight obstetrical ultrasonography practices across the Grenoble metropolitan area between 2014 and 2017 (Lyon‐Caen et al., 2019). Inclusion criteria were being under 19‐weeks pregnant at inclusion, fluent in French, and older than 18 years old, having a singleton pregnancy, being affiliated to the French national security system, planning to deliver in one of four maternity clinics in Grenoble, and living in the study area. There was a 21% participation rate amongst those approached and eligible for inclusion, resulting in a sample of 484 expectant mothers. The study area includes both urban and rural zones, and has a rather high education level compared to the average French general population due to the presence of a large research and engineering community. Compared to pregnant women in France, included SEPAGES mothers had higher rates of employment (92.9% compared to 68.1% in the general population), level of education (83% with an undergraduate or graduate degree vs. 55.4%) and French nationality (94.7% vs. 85.9%) and were more likely to be in a relationship (99.8% vs. 81.9%) (Ballard & Davies, 1996). Whilst fathers’ participation was not mandatory, the fathers of all expected children were offered to participate in the cohort. Inclusion criteria for fathers were being at least 18 years old and having social security. 410 fathers agreed to participate in the study and were eligible for inclusion. Our analyses focused on all 301 father‐child dyads in which fathers’ mental health data was available within the study period.

Every year from inclusion onwards, questionnaires, interviews and clinical examinations were used to collect sociodemographic, environmental and medical information on the children and their parents, both during and after pregnancy (von Elm et al., 2007). Both mothers and fathers of the expected child signed an informed consent form for themselves and their child prior to inclusion. Ethics board approvals were obtained from the Commission Nationale de l'Informatique et des Libertés, the Comité de Protection des Personnes Sud‐Est V, and the Agence nationale de sécurité du médicament et des produits de santé.

## 3 MEASURES

### 3.1 Indicators of PMH

Fathers’ mental health was measured during their coparent's pregnancy (mean response period 7.8 ± 5.6 weeks before birth; *n* = 166) and postpartum. For postpartum measures, fathers responded at varying time‐points in relation to their child's birth, and were thus grouped based on the timing of assessment, forming two waves: one for responses between 0 and 12 months (4.3 ± 3.7 months; *n* = 117) and one from 12–24 months (16.9 ± 3.4 months; *n* = 207). Of all included fathers (*n* = 301),112 had data at only one given time‐point, and the remaining 189 at two time‐points. At all waves, mental health was measured using the Hospital Anxiety and Depression Scale (HADS). The HADS is a self‐report measure for anxiety and depression in the general population. Based on 14 questions, responses are summed to calculate sub‐scores for the discrete depression and anxiety scales (7 items each). In cases where there were one or less missing values for either anxiety and/or depression scores, missing values were replaced by the individual's most frequently occurring answer for each respective score. Where more than one missing value for either the anxiety or depression score was present, the case was removed. Out of all 493 families, 183 were excluded for having no paternal HADS data available at any given timepoint (von Hippel, 2007). Amongst the remaining 301 fathers, out of the total 490 instances of available data (166 during pregnancy; 117 at 1 year and 207 at 2 years), 23 (4.7%) had one missing item which was then imputed, in alignment with previous analyses on the SEPAGES cohort. There were no instances of > 1 missing items. For reporting the prevalence, cut‐off scores of ≥ 8 for depression or anxiety were used (Olssøn et al., 2005). For all other analyses, scores were treated as continuous.

Cronbach alpha (α) of the HADS within our sample was .897 at pregnancy; .841 between 0–12 months and .877 12–24 months postpartum, indicating good internal consistency at all time points. Intraclass Correlation Coefficients (ICC) between the three timepoints were .73, .76 and .69 for anxiety and .57, .73 and .68 for depression, indicating good to average reliability (Gamer et al., 2005).

### 3.2 Indicators of child development

Child development was assessed at both two and three years of age, using clinical assessments performed by trained SEPAGES fieldworkers, including trained neuropsychologists, as well as validated questionnaires completed by the parents.

At two years, the child behavior checklist (CBCL/1.5‐5) was used (Achenbach, 1983; Achenbach & Ruffle, 2000). The CBCL/1.5‐5 consists of 100 items, completed by the parents. Intended to assess the emotional and behavioral problems of children from 1.5 to 5 years, it is divided into seven subscales: *(1) Emotional reactivity (2) Anxious/depressed symptoms (3) Somatic complaints (4) Withdrawn behavior (5) Sleep problems (6) Attention problems and (7) Aggressive behavior* subscales.

At three years, several measures were available. Firstly, the Behavior Rating Inventory of Executive Function, Preschool version (BRIEF‐P) (Gioia et al., 2003). The BRIEF‐P is a 63‐item, Likert‐scale questionnaire, completed by the parents. Intended to evaluate executive function, it is grouped into five non‐overlapping scales: (*1) Inhibition: (2) Shift (3) Emotional control (4) Working memory (5) Plan/Organise*.

Secondly, the social responsiveness scale (SRS) was used. The SRS is a 65‐item, Likert‐scale questionnaire completed by the parents (Bruni, 2014). Intended to screen social behavior and autism spectrum disorders (ASD), it is grouped into five scales: *(1) Social Awareness (2) Social Cognition (3) Social Communication (4) Social Motivation (5) Restricted Interests and Repetitive Behavior (RRB)*.

Finally, the Wechsler Primary and Preschool Scales of Intelligence (WPPSI) was also used (Wechsler, 2013). The WPPSI is a cognition test performed by a neuropsychologist. It consists of five verbal and five performance (nonverbal) subtests, with scores standardized post‐examination based on child age. Three main indexes can be derived from the age‐standardized scores: *(1) Verbal comprehension: (2) Visual Spatial (3) Working memory*. Within SEPAGES, a “psychologist effect” (shift in the score distributions based on the psychologist) was tested and confirmed for the visuospatial and the working memory indexes. For consistency, all three indexes were standardized for the psychologist effect using a 2‐step approach described in Mortamais et al. (Mortamais et al., 2012).

For each of the indicated child developmental measures, missing data was handled according to instrument‐specific recommended guidelines. The overall reliability and internal consistency (Cronbach α) of the CBCL was .89, .943 for the BRIEF‐P, .886 for the SRS and .852 for the WPPSI, indicating good to excellent internal consistency and reliability across all scales.

### 3.3 Confounding variables

All the following confounders were considered for inclusion in statistical analysis: Fathers’ and mothers’ age at conception, ethnicity (White/other), weight, and education level (Baccalaureate + 5 and more; Baccalaureate + 3–4 years; Baccalaureate or equivalent diploma and under), and mothers’ professional status at first trimester (employed/unemployed). Included pregnancy and child specific variables were maternal parity, prenatal tobacco (> 1 cigarette per day; yes/no) and/or alcohol consumption (no; one or less drinks per month; over one drink per month), complications during pregnancy (yes/no), gestational duration, delivery mode (vaginal/caesarean), child weight, child sex (male/female), number of weeks breastfed until week 48, and number of child hospitalizations between birth and 1 year. The main mode of childcare at 6 months was also considered (collective care (nursery, crèche, family day care, or day care centers) vs. other (spouse, partner, family members, childminders, neighbor, unlicensed nanny etc.)) alongside the arrival of subsequent siblings (yes/no).

As an additional indicator of SEP, mother and father's respective professional skill class were calculated based on International Standard Classification of Occupations (ISCO) categories (International Labour Office, 2012), grouped into four skill groups: (1) Skill level 1: Elementary occupations; (2) Skill level 2: Clerical support workers, service and sales workers, agricultural, forestry and fishery workers, craft and related trade workers, plant and machine operators and assemblers; (3) Skill levels 3–4: Managers, professionals, technicians and associate professionals; and (4) All armed forces occupations.

Maternal mental health (MMH) was measured in the third trimester (T3) and 1‐year following birth using the Hospital Anxiety and Depression Scale (HADS). As with paternal measures, anxiety and depression sub‐scores were calculated respectively, and missing data within each scale treated the same as that of fathers. For analyses, all scores were kept as continuous.

Due to lack of variation within the study population, and thus insufficient numbers within groups, mother and father relationship status; and employment status at third trimester and 1‐year postpartum were excluded from analyses. 100% of mothers were in a relationship with their child's father during pregnancy, and 94%–98% of fathers in employment at both data collection periods.

### 3.4 Data analysis

Prior to analyses, chi‐square and unpaired t‐tests were used to compare fathers’ demographics with those of excluded participants, as well as all child developmental outcome subscales.

Using an outcome‐wide analytic approach, linear regression models were used to test the association between PMH and child development (VanderWeele, 2017). In comparison to traditional regression analyses, an outcome‐wide approach permits investigation of the effects of a single exposure on multiple outcomes simultaneously, potentially reducing reporting bias and increasing statistical power through an increased chance of detecting both beneficial and harmful effects of an exposure (Anguita‐Ruiz et al., 2023). For all analyses, both depression and anxiety were used respectively as PMH exposures. To counteract collider stratification bias, a common set of confounding factors was used for all models (VanderWeele, 2019)—meaning that all models were adjusted for any variable significantly (5%) associated with at least one exposure or outcome. For outcome wide analyses, confounding variables must occur before both the exposure and outcome. Thus, all confounders occurring postnatally were only included in models using postnatal PMH as the exposure. For all models, associations were tested both non‐adjusted and adjusted for confounding variables. To control for multiple outcome bias, the significance threshold of *p*‐values was subsequently adjusted using a Hochberg correction (Andrade, 2019). Correction for multiple outcome bias was based on assessment year, with child outcomes at two and three years considered respectively and corrected as such. Missing data for confounders was imputed using the MICE package (Buuren & Groothuis‐Oudshoorn, 2011). Before imputation, 4.7% of data was missing across all variables considered for confounding. Full information on missing data is available in Table S1.

Moderation analyses were then used to test possible variation of the association between PMH and child developmental outcomes according to the three indicators previously outlined: MMH, child sex and parents’ professional skill class (ISCO category). Separate linear regression models were used to test moderation by including an interaction term (moderator*exposure), taking a *p*‐value of < .05 to represent a statistically significant interaction following adjustment using a Hochberg correction. For models using prenatal PMH, maternal HADS scores during pregnancy were used. For all postpartum PMH models, maternal postpartum HADS scores were used. For both mother and father's respective professional skill class, only two skill groups ((Stein et al., 2014) and (Kane & Garber, 2004; Velders et al., 2011)) were retained in moderator analysis, due to low representation within the other two groups (two fathers in Group 1, two in the armed forces; no mothers in either group).

All analyses were conducted using R version 4.3.2, notably with the use of packages *moments* (Komsta & Novomestky, 2022), *naniar* (Tierney & Cook, 2023)*, dplyr* (Wickham et al., 2021)*, stargazer* (Hlavac, 2022) and *sjPlot* (Lüdecke, 2025).

## 4 RESULTS

### 4.1 Descriptives

The demographic information of our study population is shown in Table 1, as well as comparison with those excluded from our analyses in Table S2. No significant difference between included and non‐included families was seen for any demographic variable except mothers’ level of education and professional skill class. Mothers of included children were more likely to be higher educated and of a higher skill level (90.7% skill level 3–4) than excluded participants (81.9%).

**TABLE 1:** Father‐child demographics, mental health data and developmental outcomes of the included study population. *SEPAGES cohort*.

		Study population	
		(*n* = 301)	
		*n*	%
**Child sex assigned at birth**	*Female*	139	46.2%
	*Male*	162	53.8%
**Father's age at inclusion, years**	*Mean (SD)*	35.0 (5.1)	
**Father's ethnicity**	*White*	290	96.3%
	*Other*	11	3.7%
**Father's level of education**	*Baccalaureate +5 and more*	185	64.5%
	*Baccalaureate +3 to 4 years*	48	16.7%
	*Baccalaureate or lower*	54	18.8%
**Father employment status—T2**	*Employed*	269	93.7%
	*Unemployed (inc. paternity or sick leave)*	18	6.3%
**Father employment status—T3**	*Employed*	241	97.9%
	*Unemployed (inc. paternity or sick leave)*	13	6.2%
**Father professional skill category** ^*^	*Skill level 3–4*	242	85.8%
	*Skill level 2*	36	12.8%
	*Skill level 1*	2	.7%
	*Armed forces*	2	.7%
**Father's weight (kg) ‐ 1 year**	*Mean (SD)*	74.0 (10.3)	
**Mother relationship status‐ T1**	*In relationship with father*	294	100.0%
	*Not in relationship with father*	0	.0%
**Mother's age at conception, years**	*Mean (SD)*	32.7 (3.7)	
**Mother's ethnicity**	*White*	289	97.0%
	*Other*	9	3.0%
**Mother's level of education**	*Baccalaureate +5 and more*	181	60.5%
	*Baccalaureate +3 to 4 years*	75	25.1%
	*Baccalaureate or lower*	43	14.4%
**Mother employment status—T1**	*Employed*	258	87.8%
	*Unemployed*	36	12.2%
**Mother employment status ‐ 1 year**	*Employed*	200	95.7%
	*Unemployed*	9	4.3%
**Mother professional skill category**	*Skill level 3–4*	264	90.7%
	*Skill level 2*	27	9.3%
	*Skill level 1*	—	—
	*Armed forces*	—	—
**Mother's weight (kg)—T1**	*Mean (SD)*	63.7 (10.1)	
**Mother's tobacco consumption during pregnancy**	*1 or less cigarettes /day*	273	96.8%
	*Over 1 cigarette /day*	9	3.2%
**Mother alcohol during pregnancy**	*No*	91	30.4%
	*One or less drinks per month*	108	36.1%
	*Over one drink per month*	100	33.4%
**Mothers' prenatal anxiety score (HADS‐A, T3)**	*Mean (SD)*	6.4 (3.1)	
**Mothers' prenatal depression score (HADS‐D, T3)**	*Mean (SD)*	4.2 (2.9)	
**Mothers' postnatal anxiety score (HADS‐A, 1 year)**	*Mean (SD)*	7.2 (3.0)	
**Mothers' postnatal depression score (HADS‐D, 1 year)**	*Mean (SD)*	4.0 (2.8)	
**Diagnosed complications during pregnancy**	*No*	266	89.6%
	*Yes*	31	10.4%
**Gestational duration (weeks)** ^**^	*Mean (SD)*	39.7 (1.4)	
**Birth weight (child), g**	*Mean (SD)*	3278.2 (417.1)	
**Delivery mode**	*Vaginal*	259	86.0%
	*Caesarean*	42	14.0%
**Mother's parity at birth**	*0*	142	47.2%
	*1*	130	43.2%
	*2*	29	9.6%
**No. of weeks breastfed before week 48**	*Mean (SD)*	27.0 (15.9)	
**Number of hospitalizations between birth and 2 months old**	*0*	273	91.9%
	*1*	23	7.7%
	*2*	1	.3%
**Number of hospitalizations between 2 and 12 months old**	*0*	282	96.9%
	*1*	9	3.1%
	*2*	0	.0%
**Main mode of childcare at 6 months**	*Collective day care*	173	60.9%
	*Other*	111	39.1%
**Main mode of childcare at 12 months**	*Collective day care*	227	79.9%
	*Other*	57	20.1%
**Number of children in household (inc. part‐time)—T1**	*Mean (SD)*	.7 (.7)	
**Number of brothers and sisters ‐ 1 year**	*Mean (SD)*	.7 (.7)	

Paternal mental health			
		n	%
**Prenatal Anxiety** ^***^	*No*	121	72.9%
	*Yes*	45	27.1%
**Prenatal Depression** ^***^	*No*	121	72.9%
	*Yes*	45	27.1%
**Postnatal Anxiety** ^***^ **‐ 0–12 months**	*No*	91	77.8%
	*Yes*	26	22.2%
**Postnatal Depression** ^***^ **‐ 0–12 months**	*No*	114	97.4%
	*Yes*	3	2.6%
**Postnatal Anxiety** ^***^ **‐ 12–24 months**	*No*	154	74.4%
	*Yes*	53	25.6%
**Postnatal Depression** ^***^ **‐ 12–24 months**	*No*	154	74.4%
	*Yes*	53	25.6%

Child developmental outcomes			
		n	Mean (SD)
**Child Behavior Checklist (CBCL)**	*Emotional reactivity*	286	.5 (.3)
	*Anxiety/Depression*	286	.3 (.3)
	*Somatic complaints*	285	.4 (.3)
	*Withdrawn behavior*	286	.2 (.2)
	*Sleep problems*	286	.5 (.3)
	*Attention problems*	286	.5 (.2)
	*Aggressive behavior*	286	1.0 (.2)
	*Other problems*	286	.9 (.2)
	*Total score*	286	32.5 (14.4)
**Wechsler Primary and Preschool Scales of Intelligence (WPPSI)**	*Verbal comprehension*	265	112.2 (11.7)
	*Visuospatial index*	266	110.4 (10.6)
	*Working memory*	264	105.7 (10.7)
	*Total Intelligence Quotient (IQ)*	265	112.9 (11.0)
**Behavior Rating Inventory of Executive Function, Preschool version (BRIEF‐P)**	*Inhibitory control and impulsivity*	279	4.9 (.5)
	*Shift*	284	1.1 (.1)
	*Emotional control*	284	3.8 (.5)
	*Working memory*	277	1.3 (.1)
	*Planning/organization*	282	3.7 (.4)
**Social Responsiveness Scale (SRS)**	*Social Awareness*	280	5.8 (2.7)
	*Social Cognition*	280	2.4 (.7)
	*Social Communication*	280	.9 (.2)
	*Social Motivation*	280	.8 (.2)
	*Repetitive Behavior (RRB)*	280	.6 (.3)
	*SRS Total*	280	1.4 (.2)

Abbreviations: T1 /T2/T3 = First/Second/Third trimester.

^*^Based on ISCO scoring. Skill level 1: Elementary occupations; Skill level 2: Clerical support workers, service and sales workers, agricultural, forestry and fishery workers, craft and related trade workers, plant and machine operators and assemblers; Skill levels 3‐–4: Managers, professionals, technicians and associate professionals.

^**^Calculated from mother's last menstrual period

^***^Hospital Anxiety and Depression Scale (HADS); cut‐off score of 8.

Based on clinical HADS cut‐offs, 27% (*n* = 45) of fathers experienced anxiety prenatally, 22% (*n* = 26) at 0–12 months postpartum and 26% (*n* = 53) at 12–24 months postpartum. 27.1% of fathers experienced depression prenatally, 2.6% at 0–12 months postpartum and 26% at 12–24 months postpartum.

Regarding measures of child development, no significant difference amongst scores was observed between included and excluded children except for the aggressive behavior subscale of the CBCL, with excluded children's scores ranging more.

### 4.2 PMH and child development

The association between prenatal PMH and child development was nonsignificant for all measures of child development (Tables 2a, 2b, 2c), both before and after adjusting for confounding factors.

**TABLE 2a:** The effect of prenatal (7.8 ± 5.6 weeks before birth; *n* = 166) paternal mental health on child development. Linear regression modelling, both non‐adjusted and adjusted for confounding variables. *β* = unstandardized coefficient, SE = Standard error, *p*_Adj _= *p* value adjusted for multiple outcomes (Hochberg correction).

		Pregnancy							
		Non‐adjusted				Adjusted			
		Paternal anxiety*		Paternal depression*		Paternal anxiety*		Paternal depression*	
		*β* (SE)	*p / p*_Adj_	*β* (SE)	*p / p*_Adj_	*β* (SE)	*p / p*_Adj_	*β* (SE)	*p / p*_Adj_
**CBCL**	*Emotional reactivity*	−.01 (.04)	.81 / .97	.02 (.08)	.84 / .96	0 (.02)	.82 / .82	0 (.02)	.95 / .95
	*Anxiety/Depression*	0 (.04)	.97 / .97	0 (.07)	.96 / .96	.01 (.02)	.44 / .82	.01 (.02)	.34 / .95
	*Somatic complaints*	.04 (.04)	.25 / .97	.09 (.07)	.24 / .96	.02 (.02)	.02 / .16	.01 (.02)	.16 / .95
	*Withdrawn behavior*	.03 (.03)	.37 / .97	.06 (.06)	.28 / .96	.01 (.02)	.29 / .82	.01 (.02)	.22 / .95
	*Sleep problems*	.02 (.04)	.67 / .97	.1 (.08)	.20 / .96	.01 (.02)	.30 / .82	.01 (.02)	.13 / .91
	*Attention problems*	.01 (.03)	.80 / .97	−.01 (.07)	.91 / .96	0 (.02)	.62 / .82	0 (.02)	.80 / .95
	*Aggressive behavior*	.02 (.03)	.57 / .97	.11 (.06)	.06 / .48	0 (.01)	.70 / .82	.01 (.02)	.08 / .64
	*Other problems*	−.01 (.02)	.68 / .97	.06 (.05)	.24 / .96	0 (.01)	.78 / .82	.01 (.02)	.27 / .95
**WPPSI**	*Verbal comprehension index*	1.23 (1.8)	.49 / .97	−.26 (3.69)	.94 / .96	.31 (.72)	.40 / .91	.2 (.81)	.63 / .95
	*Visuospatial index*	−2.08 (1.6)	.19 / .97	−1.42 (3.3)	.67 / .96	−.5 (.67)	.14 / .91	−.26 (.75)	.50 / .95
	*Working memory index*	.92 (1.64)	.58 / .97	.38 (3.36)	.91 / .96	.24 (.68)	.49 / .91	−.08 (.76)	.84 / .95
**BRIEF‐P**	*Inhibitory control and impulsivity*	−.08 (.07)	.29 / .97	−.08 (.15)	.59 / .96	−.01 (.03)	.40 / .91	0 (.03)	.95 / .95
	*Shift*	−.02 (.01)	.13 / .97	.01 (.03)	.83 / .96	0 (.01)	.45 / .91	0 (.01)	.81 / .95
	*Emotional control*	−.02 (.06)	.80 / .97	.1 (.12)	.42 / .96	−.01 (.03)	.63 / .91	.02 (.03)	.23 / .95
	*Working memory*	−.02 (.01)	.18 / .97	−.04 (.02)	.13 / .96	0 (.01)	.31 / .91	0 (.01)	.27 / .95
	*Planning/organization*	−.07 (.05)	.19 / .97	−.05 (.11)	.64 / .96	−.01 (.02)	.27 / .91	−.01 (.03)	.45 / .95
**SRS**	*Social Awareness*	−.01 (.37)	.97 / .97	1 (.76)	.19 / .96	−.06 (.16)	.46 / .91	.15 (.17)	.09 / .95
	*Social Cognition*	.04 (.09)	.70 / .97	−.07 (.2)	.71 / .96	0 (.04)	.91 / .91	−.01 (.04)	.67 / .95
	*Social Communication*	−.01 (.03)	.81 / .97	0 (.06)	.96 / .96	0 (.01)	.83 / .91	0 (.02)	.62 / .95
	*Social Motivation*	−.02 (.03)	.56 / .97	.06 (.07)	.41 / .96	0 (.02)	.57 / .91	0 (.02)	.65 / .95
	*Repetitive Behavior (RRB)*	.02 (.04)	.57 / .97	.1 (.08)	.21 / .96	.01 (.02)	.45 / .91	.02 (.02)	.02 / .26

^*^Hospital Anxiety and Depression Scale (HADS).

Abbreviations: BRIEF‐P, Behavior Rating Inventory of Executive Function, Preschool version; CBCL, Child Behavior Checklist; SRS, Social Responsiveness Scale; WPPSI, Wechsler Primary and Preschool Scales of Intelligence.

**TABLE 2b:** The effect of postnatal (0–12 months; *n* = 117) paternal mental health on child development. Linear regression modelling, both non‐adjusted and adjusted for confounding variables. *β* = unstandardized coefficient, SE = Standard error, *p*_Adj_ = *p* value adjusted for multiple outcomes (Hochberg correction).

		0–12 months							
		Non‐adjusted				Adjusted			
		Paternal anxiety*		Paternal depression*		Paternal anxiety*		Paternal depression*	
		*β (SE)*	*p / p*_Adj_	*β (SE)*	*p / p*_Adj_	*β (SE)*	*p / p*_Adj_	*β (SE)*	*p / p*_Adj_
**CBCL**	*Emotional reactivity*	−.03 (.05)	.54 / .84	−.02 (.05)	.71 / .91	−.01 (.02)	.33 / .98	0 (.02)	.81 / .99
	*Anxiety/Depression*	−.07 (.05)	.15 / .84	−.02 (.05)	.64 / .91	0 (.02)	.65 / .98	0 (.02)	.67 / .99
	*Somatic complaints*	.05 (.05)	.25 / .84	−.01 (.05)	.80 / .91	.01 (.02)	.27 / .98	.01 (.02)	.42 / .99
	*Withdrawn behavior*	.08 (.03)	.02 / .16	.05 (.03)	.12 / .91	.01 (.01)	.16 / .98	.01 (.01)	.13 / .99
	*Sleep problems*	−.05 (.06)	.41 / .84	−.03 (.05)	.61 / .91	0 (.02)	.75 / .98	0 (.02)	.57 / .99
	*Attention problems*	−.02 (.04)	.66 / .84	0 (.04)	.91 / .91	0 (.01)	.98 / .98	0 (.02)	.57 / .99
	*Aggressive behavior*	−.01 (.04)	.84 / .84	−.01 (.04)	.86 / .91	0 (.01)	.71 / .98	0 (.02)	.46 / .99
	*Other problems*	−.02 (.04)	.64 / .84	−.02 (.04)	.66 / .91	−.01 (.01)	.21 / .98	0 (.01)	.99 / .99
**WPPSI**	*Verbal comprehension index*	.09 (2.15)	.97 / .98	−.77 (2.08)	.71 / .99	.05 (.59)	.88 / .88	−.32 (.66)	.33 / .88
	*Visuospatial index*	1.55 (1.94)	.42 / .98	−.03 (1.88)	.99 / .99	.18 (.58)	.53 / .88	.05 (.65)	.88 / .88
	*Working memory index*	1.51 (1.9)	.43 / .98	−1.58 (1.83)	.39 / .99	.18 (.59)	.55 / .88	−.24 (.66)	.48 / .88
**BRIEF‐P**	*Inhibitory control and impulsivity*	.04 (.1)	.69 / .98	.13 (.1)	.19 / .99	.01 (.03)	.45 / .88	.01 (.03)	.39 / .88
	*Shift*	.01 (.02)	.56 / .98	.03 (.02)	.08 / .99	0 (.01)	.82 / .88	0 (.01)	.86 / .88
	*Emotional control*	−.03 (.09)	.75 / .98	.06 (.08)	.47 / .99	.01 (.03)	.54 / .88	0 (.03)	.72 / .88
	*Working memory*	.01 (.02)	.55 / .98	.01 (.02)	.69 / .99	0 (.01)	.27 / .88	0 (.01)	.44 / .88
	*Planning/organization*	.06 (.06)	.37 / .98	.08 (.06)	.21 / .99	.01 (.02)	.44 / .88	.01 (.02)	.45 / .88
**SRS**	*Social Awareness*	−.01 (.49)	.98 / .98	.47 (.47)	.32 / .99	.01 (.14)	.84 / .88	.12 (.15)	.12 / .88
	*Social Cognition*	.1 (.13)	.43 / .98	.18 (.12)	.13 / .99	.01 (.03)	.48 / .88	.04 (.04)	.02 / .24
	*Social Communication*	−.04 (.05)	.41 / .98	.01 (.05)	.80 / .99	0 (.01)	.82 / .88	.01 (.01)	.08 / .88
	*Social Motivation*	.06 (.04)	.17 / .98	.04 (.04)	.34 / .99	.01 (.02)	.35 / .88	.01 (.01)	.11 / .88
	*Repetitive Behavior (RRB)*	−.03 (.06)	.61 / .98	−.02 (.05)	.66 / .99	.01 (.02)	.36 / .88	.02 (.02)	.02 / .24

^*^Hospital Anxiety and Depression Scale (HADS).

Abbreviations: BRIEF‐P, Behavior Rating Inventory of Executive Function, Preschool version; SRS, Social Responsiveness Scale; CBCL, Child Behavior Checklist; WPPSI, Wechsler Primary and Preschool Scales of Intelligence.

**TABLE 2c:** The effect of postnatal (12–24 months; *n* = 207) paternal mental health on child development. Linear regression modelling, both non‐adjusted and adjusted for confounding variables. *β* = unstandardized coefficient, SE = Standard error, *p*_Adj_ = *p* value adjusted for multiple outcomes (Hochberg correction).

		12–24 months							
		Non‐adjusted				Adjusted			
		Paternal anxiety*		Paternal depression*		Paternal anxiety*		Paternal depression*	
		*β (SE)*	*p / p*_Adj_	*β (SE)*	*p / p*_Adj_	*β (SE)*	*p / p*_Adj_	*β (SE)*	*p / p*_Adj_
**CBCL**	*Emotional reactivity*	−.01 (.03)	.71 / .99	−.01 (.03)	.73 / .94	0 (.03)	.74 / .8	0 (.03)	.98 / .98
	*Anxiety/Depression*	−.01 (.03)	.71 / .99	0 (.03)	.94 / .94	−.01 (.02)	.3 / .8	.01 (.03)	.56 / .98
	*Somatic complaints*	.03 (.03)	.36 / .99	.01 (.03)	.65 / .94	.01 (.02)	.44 / .8	0 (.03)	.91 / .98
	*Withdrawn behavior*	.04 (.02)	.12 / .96	.03 (.02)	.17 / .94	.02 (.02)	.01 / .08	.01 (.02)	.18 / .98
	*Sleep problems*	−.01 (.03)	.75 / .99	−.02 (.03)	.6 / .94	−.02 (.03)	.19 / .8	0 (.03)	.92 / .98
	*Attention problems*	0 (.03)	.99 / .99	−.02 (.03)	.5 / .94	−.01 (.02)	.56 / .8	.01 (.03)	.58 / .98
	*Aggressive behavior*	.01 (.03)	.83 / .99	.02 (.02)	.45 / .94	0 (.02)	.8 / .8	0 (.02)	.97 / .98
	*Other problems*	−.02 (.03)	.45 / .99	−.01 (.03)	.79 / .94	−.01 (.02)	.55 / .8	0 (.02)	.9 / .98
**WPPSI**	*Verbal comprehension index*	−1.51 (1.47)	.30 / .98	−1.57 (1.39)	.26 / .94	.71 (.91)	.12 / .96	.58 (1.12)	.3 / .74
	*Visuospatial index*	.18 (1.4)	.90 / .98	.47 (1.32)	.72 / .94	.55 (.86)	.21 / .96	.21 (1.06)	.69 / .74
	*Working memory index*	.04 (1.39)	.98 / .98	−1.15 (1.32)	.38 / .94	.42 (.87)	.33 / .96	−.23 (1.06)	.66 / .74
**BRIEF‐P**	*Inhibitory control and impulsivity*	.03 (.06)	.59 / .98	.02 (.06)	.75 / .94	0 (.05)	.96 / .96	.03 (.06)	.26 / .74
	*Shift*	0 (.01)	.96 / .98	−.01 (.01)	.5 / .94	0 (.01)	.38 / .96	.01 (.01)	.03 / .39
	*Emotional control*	.04 (.05)	.48 / .98	.01 (.05)	.78 / .94	−.01 (.04)	.68 / .96	.03 (.05)	.26 / .74
	*Working memory*	0 (.01)	.79 / .98	0 (.01)	.94 / .94	0 (.01)	.23 / .96	.01 (.01)	.21 / .74
	*Planning/organization*	.02 (.05)	.67 / .98	.02 (.04)	.6 / .94	.01 (.03)	.38 / .96	.02 (.04)	.19 / .74
**SRS**	*Social Awareness*	−.03 (.33)	.93 / .98	.39 (.31)	.21 / .94	.06 (.23)	.59 / .96	.26 (.28)	.06 / .72
	*Social Cognition*	.04 (.08)	.65 / .98	.09 (.08)	.27 / .94	.02 (.06)	.58 / .96	.06 (.07)	.1 / .74
	*Social Communication*	−.01 (.03)	.61 / .98	.02 (.03)	.52 / .94	0 (.02)	.86 / .96	.01 (.03)	.29 / .74
	*Social Motivation*	.01 (.03)	.79 / .98	.03 (.03)	.35 / .94	.02 (.02)	.06 / .78	.02 (.03)	.21 / .74
	*Repetitive Behavior (RRB)*	−.01 (.04)	.80 / .98	.03 (.03)	.4 / .94	0 (.03)	.8 / .96	.01 (.03)	.74 / .74

^*^Hospital Anxiety and Depression Scale (HADS).

Abbreviations: BRIEF‐P, Behavior Rating Inventory of Executive Function, Preschool version; SRS, Social Responsiveness Scale; CBCL, Child Behavior Checklist; WPPSI, Wechsler Primary and Preschool Scales of Intelligence.

The same was seen for postnatal PMH occurring between 0–12 months and 12–24 months, both before and after adjusting for confounding factors.

### 4.3 Moderation effects

#### 4.3.1 Maternal anxiety

Following corrections for multiple testing, maternal anxiety was not seen to significantly moderate the relationship between PMH and child development for any outcome, out of a possible 126 (six PMH exposures; 21 developmental outputs) (Table S3).

#### 4.3.2 Maternal depression

Maternal depression (MD) was seen to moderate the influence of PMH for two associations, following correction for multiple outcomes. Both instances were in relation to prenatal paternal anxiety and prenatal MD. A significant moderation effect was seen by MD on the relationship between prenatal paternal anxiety and the BRIEF‐P working memory subscale (*β* = .17; SE = .04; *p*_Adj _< .*01*) and the BRIEF‐P plan/organize subscale (*β* = .09; SE = .03; *p*_Adj _< .*05*), with children exposed to higher prenatal paternal anxiety showing worse working memory outcomes (higher scores) and abilities to plan and organize current and future‐oriented tasks when also exposed to higher prenatal MD scores.

#### 4.3.3 Child sex

Child sex was not seen to significantly moderate the relationship between PMH and child development for any outcome, for all three PMH timepoints (prenatal; 0–12 months; 12–24 months).

#### 4.3.4 ISCO Professional class

Fathers’ professional class (ISCO score) was not seen to significantly moderate the relationship between PMH and child development for any outcome, at all three time points.

Mothers’ professional class (ISCO score) was seen to be the most significant moderator, moderating the influence of PMH for four associations following correction for multiple outcomes. For prenatal paternal anxiety, moderation by mothers’ ISCO was seen for the CBCL attention problems (*β* = .37; SE = .13; *p*_Adj _< .05), BRIEF‐P working memory (*β* = .13; SE = .05; *p*_Adj _< .05), and SRS social cognition (*β* = 1.24; SE = .38; *p*_Adj _< .05) subscales. Mother's ISCO score also moderated the association of paternal anxiety at 12–24 months with the WPPSI verbal comprehension index (β = ‐32.89; SE = 10.78; *p*_Adj_ *<* .05).

Amongst children whose mothers had lower ISCO scores, higher paternal anxiety scores associated with higher attention problem scores, poorer working memory and lower visuospatial scores, but greater social cognition.

## 5 DISCUSSION

This is the first study to examine associations between both paternal depression and anxiety on child socioemotional, behavioral, and cognitive development at two and three years of age in an outcome‐wide analysis. The rates of both depression (3%–27%) and anxiety (22%–27%) amongst fathers were within expected global and French ranges (Ballard & Davies, 1996; Philpott et al., 2019; Paulson & Bazemore, 2010; Nakamura et al., 2020). Neither paternal anxiety nor depression associated with any studied measure of child development. Notably, whilst neither child sex nor paternal professional class moderated this relationship, we observed that MMH and professional class significantly moderated the association between paternal anxiety and several aspects of child development.

### 5.1 Association of PMH to child development

Though inconsistent throughout the literature, significant associations have been found between PMH (PMH) and child development (Scarlett et al., 2023). Despite this being the first outcome wide analysis between PMH and child development the overall nonsignificant associations in our study were thus unexpected. Associations between PMH and child development have been seen as early as 2‐months (Spry et al., 2020; Sweeney & MacBeth, 2016), with numerous reviews finding the association between PMH and child development to be stronger in younger children of ages similar to that within our study (Stein et al., 2014; Cui et al., 2020). Regarding our choice of outcomes, studies have shown PMH to impact similar measures of child development, such as ADHD at three years (Breaux et al., 2017), and social withdrawal at 4–18 months (Mäntymaa et al., 2008).

However, it is important to note that within our included families, 94%–98% of fathers and 88%–96% of mothers were in employment, as well as 76%–81% with a university degree or higher. When compared to the rate of employment within the French general population (68%), this indicates a considerably high SEP amongst SEPAGES participants. Low SEP has been seen to accentuate the adverse child outcomes that associate with PMH (Stein et al., 2014; Cui et al., 2020). Our findings might then indicate that the effects of PMH on child development are more pertinent for less privileged families/fathers, with the demographics of our sample leading to an underestimation of the relationship between PMH and child development. In support of this, one study (Narayanan & Nærde, 2016) found the impact of fathers’ depression on child behavioral problems at 48‐months disappeared once controlling for socioeconomic factors. The high rate of employment amongst our sample may have also impacted the frequency of paternal child care, which has been shown to moderate the impact of PMH on child development (Mezulis et al., 2004; Sweeney & MacBeth, 2016; Gutierrez‐Galve et al., 2015). However, it is important to note that many forms of employment exist, such as father's working from home, which would not necessarily result in father's spending more time away from the home environment itself. Whilst we did not have data on this within the SEPAGES cohort, it should be considered in future studies, particularly in lieu of the evolution of working environments following the COVID‐19 pandemic (Adrjan et al., 2025).

Interestingly, one study saw that amongst a sample of high‐SEP fathers, greater paternal depression and anxiety associated with higher child IQ and fewer behavioral difficulties (Jones et al., 2023). The lack of association seen within our sample might thus result from similar positive associations existing within certain father‐child dyads, thus negating the expected negative association amongst others and resulting in an overall non‐significance. A possible explanation for this may be certain positive outcomes on paternal involvement or parenting behaviors that might associate with depression or anxiety; such as more time spent at home (Köse & Namjoo, 2024). Indeed, qualitative reports reveal stay‐at‐home fathers often feel that spending more time at home has positively impacted their relationship with their children (Davis et al., 2020). It is also worth noting that Jones and colleagues’ aforementioned study (2023) also relied on a particularly high SEP sample, as did ours. It is thus possible that both of our findings, which contrast the majority of the existing literature, may be linked to our sample demographics.

### 5.2 Moderating factors

The most significant moderator examined in our study was parent's professional class, a key indicator of SEP. However, moderation was seen exclusively for mother's professional class. This may partially stem from the gender roles surrounding parenting responsibilities. Despite ongoing changes, mothers remain overall more present than fathers in their children's upbringing, showing greater parenting responsibilities (Renk et al., 2003) and time commitment (Ballard & Davies, 1996; Craig, 2006). Thus, their professional situation and associated factors may carry greater weight on children's developmental outcomes. Our findings further suggest that the combination of paternal anxiety, as opposed to depression, and lower maternal professional class was the most detrimental to child cognitive and behavioral outcomes. This may be due to changes in the mutual parenting dynamic. Lower ISCO scores associate with greater constraints relative to working hours (Choi et al., 2020), which may in turn impede the duration or quality of parental involvement. The reduced maternal involvement associated with lower ISCO scores may therefore expose children more to their father's anxiety and associated changes to parenting behaviors. Indeed, children of parents with lower ISCO scores showed primarily worse developmental outcomes when exposed to higher paternal anxiety. Anxiety in fathers has been seen to associate with more intrusive and controlling parenting styles, alongside increased rates of forceful discipline (Teetsel et al., 2014; Möller et al., 2016). Studies also show that parents with more stimulating and autonomous jobs use less harsh or restrictive parenting, opting for a more positive, warm and responsive approach (Greenberger et al., 1994; Costigan & Cox, 2003; Mason et al., 1994; Whitbeck et al., 1997). Warm and responsive parenting can foster secure attachment and executive functioning during early child development (Kong & Yasmin, 2022; Waller et al., 2014), whilst intrusive or inconsistent interactions may undermine emotion regulation and hinder cognitive skills like working memory (Jiang et al., 2023; Mortensen & Barnett, 2019). The predicted changes in maternal parenting style associated with lower ISCO scores may therefore act in conjunction with those associating with paternal anxiety, exerting a greater cumulative effect on their child's home environment.

Except for two subscales (working memory and planning/organization) at 3 years, neither maternal depression nor anxiety appeared to moderate the relationship between PMH and child development. This overall non‐significance of MMH is particularly interesting, as numerous studies show PMH to be an important moderator of the effect of MMH on offspring development (Connell & Goodman, 2002; Dietz et al., 2009; Letourneau et al., 2019). Our finding may potentially be due to the overall stability of child development seen across results. Once again, the SEP of our sample may have acted as a protective factor, as the influence of MMH on child development has been repeatedly found to depend partially on family SEP (Passareli‐Carrazzoni et al., 2018; Smith et al., 2023; Clément et al., 2024). Finally, child sex did not appear to moderate the relationship between PMH and child development. The current literature available on child sex in relation to the effects of PMH is mixed and varies between reviews (Cheung & Theule, 2019; Connell & Goodman, 2002), suggesting there may be better predictors of child outcomes in relation to PMH than child sex.

## 6 STRENGTHS AND LIMITATIONS

This study's outcome‐wide framework allowed for a more comprehensive understanding of the relationship between these outcomes and PMH than traditional single‐outcome models. Whilst our results were primarily non‐significant, there were a few marginally significant associations prior to adjusting for multiple outcomes. This demonstrates the value of an outcome‐wide approach, as it prevents the selective reporting of several marginally significant findings, which would have thus misrepresented of the relationship between PMH and child development seen across our sample.

In using four validated measures of child development, we investigated a large range of child outcomes, including potential indicators of autism and attention deficit disorder. SEPAGES questionnaires provided numerous, detailed waves of potential pre‐ and postnatal confounders, as early as the first trimester of pregnancy. Child development was also measured at both two and three years of age, between which significant changes occur in child temperament, autonomy, gender roles, and peer relationships (Malik & Marwaha, 2024). Finally, child development was assessed by both parent self‐report and clinical psychologists. This is particularly important, as reports on child development may differ based on the objectivity of the observer (Achenbach et al., 1987). Relying solely on parent‐reports could have therefore introduced bias.

However, our sample size is relatively small and unrepresentative of the French national population (e.g. 96% White; 94%–98% in employment), thus limiting the external validity of our results. Although an outcome‐wide analytical approach is thought to increase statistical power when analyzing multiple outcomes (VanderWeele, 2017; Anguita‐Ruiz et al., 2023), partially challenging this, it is important to note that, given our sample size (301) and corrected significance thresholds, our analyses had 80% power to detect effects ranging from approximately 2.8 times the standard error SE (e.g., ∼ .1 units for outcomes with SE ≈ .04 and ∼4–5 units for outcomes with SE ≈ 1.6–1.8). Fathers’ participation was also optional, risking selection bias in favor of more engaged fathers, which has in turn been seen to associate with improved child outcomes (Rollè et al., 2019; Bennett et al., 2002). Furthermore, PMH was measured in a cross‐sectional manner, thus the duration of symptoms was not accounted for. However, the fact that measurements of PMH occurred prior to all measures of child development partially minimizes the risk of bidirectional effects. The prevalence of comorbid depression and anxiety is also important, including amongst fathers (Scarlett et al., 2024; Dennis et al., 2022), and whilst our study was concerned more so with comparing the respective impact of each, the potential cumulative impact of both warrants further investigation. Indeed, one study found increased risks of psychopathological outcomes in offspring when exposed to comorbid paternal depression and anxiety (Weissman et al., 2006). To our knowledge, there are no other studies that properly account for both conditions amongst fathers in relation to child outcomes.

Finally, data were unavailable for several key potential confounding factors known to associate with either PMH or child development, due either to the questionnaire design or homogeneity within the study cohort. However, although employment status could not be included, Perry‐Jenkins et al. (2020) argue that, when linking parental employment and children's development, the type of work may be more important than employment status itself. Thus, our inclusion of parents’ professional category instead may have permitted a more sensitive moderator analysis.

## 7 CONCLUSION

This study provides an outcome‐wide assessment of the association between pre‐ and postnatal PMH and child development at 2–3 years old, spanning numerous child outcomes. Neither fathers’ depression nor anxiety appeared to significantly associate with any measures of their child's development at both ages. However, we found that both mother's professional class and maternal depression had a moderating effect on the relationship between fathers’ anxiety and child development. Whilst the influence of MMH was less impactful than expected, our findings suggest family social class appears therefore to play an important role in the relationship between PMH and child development. These findings are especially relevant considering the number of widening disparities between children from low and high‐income households. Future research on the effect of fathers’ mental illness on their child should focus on actively recruiting participants from more diverse socioeconomic backgrounds often underrepresented in current research. Moreover, these results indicate that, whilst most studies on PMH have focused on depression, paternal anxiety might be more pertinent to child outcomes, and thus inclusion of paternal anxiety is important for future research on child development.

## FUNDING INFORMATION

The SEPAGES cohort was supported by the European Research Council (N°311765‐E‐DOHaD), the European Community's Seventh Framework Programme (FP7/2007‐206‐ N°308333‐892 HELIX), the European Union's Horizon 2020 research and innovation programme (N° 874583 ATHLETE Project, N°825712 OBERON Project), the French Research Agency ‐ ANR (PAPER project ANR‐12‐PDOC‐0029‐01, SHALCOH project ANR‐14‐CE21‐0007, ANR‐15‐IDEX‐02 and ANR‐15‐IDEX5, GUMME project ANR‐17‐CE34‐0013, ETAPE project ANR‐18‐CE36‐0005 ‐ EDeN project ANR‐19‐CE36‐0003‐01 – MEMORI project ANR 21‐CE34‐0022, ORANDANI project ANR‐22‐CE36‐0018), the French Agency for Food, Environmental and Occupational Health & Safety ‐ ANSES (CNAP project EST‐2016‐121, PENDORE project EST‐2018‐1‐264, HyPAxE project EST‐2019/1/039, PENDALIRE project EST‐2022‐169), the Plan Cancer (Canc'Air project), the French Cancer Research Foundation Association de Recherche sur le Cancer – ARC, the French Endowment Fund AGIR for chronic diseases – APMC (projects PRENAPAR, LCI‐FOT, DysCard), the French Endowment Fund for Respiratory Health, the French Fund – Fondation de France (CLIMATHES – 00081169, SEPAGES 5 – 00099903, ELEMENTUM ‐ 00124527).

## ETHICS STATEMENT

Ethics board approvals were obtained from the Commission Nationale de l'Informatique et des Libertés (CNIL, authorisation n°2014‐263, 2014‐06‐26), the CPP (Comité de Protection des Personnes Sud‐Est V, authorisation n°13‐CHUG‐44, 2014‐02‐25) and the Agence nationale de sécurité du médicament et des produits de santé (ANSM, authorisation n°2013‐A01491‐44, 2013‐12‐17).

## CONFLICT OF INTEREST STATEMENT

No conflicts of interest to disclose.

## Supporting information



Supporting Information

Supporting Information

## Data Availability

The data supporting the findings of this study are available from the SEPAGES study group upon reasonable request; available at the following URL: https://cohorte‐sepages.fr/fr/espace‐scientifique‐acces‐aux‐donnees. The analyses presented here were not preregistered, and all analytic code necessary to reproduce the analyses is available from the first author.
