# Supplementary material for: Paternal perinatal mental health and early child development: An outcome‐wide analysis
Source: Infant Ment Health J. 2026 Jun 10;47(4):e70104. doi: 10.1002/imhj.70104 (PMC13254239; doi:10.1002/imhj.70104)
Supplement: Supplementary file 1 — Supporting Information [file IMHJ-47-0-s001.docx]

| **Supplementary Table 1 -** Missing variable data within the study population (n = 301); *SEPAGES cohort.* | |
| --- | --- |
| **Variable** | **Missing** |
| Child sex at birth | 0.0% |
| Father's age at inclusion, years | 1.3% |
| Father's ethnicity | 0.0% |
| Father's nationality | 4.7% |
| Father's level of education | 4.7% |
| Father employment status - T2 | 4.7% |
| Father employment status - 1 year | 20.5% |
| Father professional skill class | 6.3% |
| Mother relationship status (T1) | 2.3% |
| Father's weight (kg) - 1 year | 19.7% |
| Mother's age at conception, years | 0.0% |
| Mother's ethnicity | 1.0% |
| Mother's level of education | 7.0% |
| Mother's ethnicity | 0.7% |
| Mother employment status - First trimester | 2.3% |
| Mother employment status - 1 year | 29.0% |
| Mother professional skill class | 3.3% |
| Mother's weight (kg) - First trimester | 0.0% |
| Mother's tobacco consumption during pregnancy | 6.3% |
| Mother alcohol during pregnancy | 0.7% |
| Mothers' prenatal mental health score (HADS, T3) | 3.0% |
| Mothers' postnatal mental health score (HADS, 1 year) | 18.1% |
| Diagnosed complications during pregnancy | 1.3% |
| Gestational duration (weeks)* | 0.0% |
| Birth weight (child), g | 0.0% |
| Delivery mode | 0.0% |
| Mother's parity at birth | 0.0% |
| No. of weeks breastfed before week 48 | 2.3% |
| Number of child hospitalisations between birth and 2 months old | 0.8% |
| Number of child hospitalisations between 2 and 12 months old | 2.7% |
| Main mode of child care at 6 months | 4.2% |
| Main mode of child care at 12 months | 4.2% |
| Number of children in household (inc. part-time) - T1 | 6.3% |
| Number of brothers and sisters - 1 year | 16.2% |
| *T1/T2/T3= First/Second/Third trimester.* | |

| **Supplementary Table 2 -** Comparison of father-child demographics, mental health data and developmental outcomes between included and excluded study populations. *SEPAGES cohort*. *P values = Chi-square and unpaired t-test.* | | | | | | |
| --- | --- | --- | --- | --- | --- | --- |
|  |  | **Study population (n = 301)** | | **Excluded sample (n = 183)** | |  |
|  |  | **n** | **%** | **n** | **%** | **p value** |
| **Child sex assigned at birth** | *Female* | 139 | 46.2% | 83 | 46.6% | 1.000 |
|  | *Male* | 162 | 53.8% | 95 | 53.4% |  |
| **Father's age at inclusion, years** | *Mean (SD)* | 35.0 (5.1) | | 35.0 (5.6) | | 0.984 |
| **Father's ethnicity** | *White* | 290 | 96.3% | 100 | 91.7% | 0.099 |
|  | *Other* | 11 | 3.7% | 9 | 8.3% |  |
| **Father's level of education** | *Baccalaureate +5 and more* | 185 | 64.5% | 4 | 57.1% |  |
|  | *Baccalaureate +3 to 4 years* | 48 | 16.7% | 1 | 14.3% |  |
|  | *Baccalaureate or lower* | 54 | 18.8% | 2 | 28.6% |  |
| **Father employment status - T2** | *Employed* | 269 | 93.7% | 11 | 100.0% | NA* |
|  | *Unemployed (inc. paternity or sick leave)* | 18 | 6.3% | 0 | 0.0% |  |
| **Father employment status - T3** | *Employed* | 241 | 97.9% | 2 | 100.0% | NA* |
|  | *Unemployed (inc. paternity or sick leave)* | 13 | 6.2% | 0 | 0.0% |  |
| **Father professional skill class*** | *Skill level 3-4* | 242 | 85.8% | 8 | 80% | NA* |
|  | *Skill level 2* | 36 | 12.8% | 1 | 10.0% |  |
|  | *Skill level 1* | 2 | 0.7% | 1 | 10.0% |  |
|  | *Armed forces* | 2 | 0.7% | 0 | 0.0% |  |
| **Father's weight (kg) - 1 year** | *Mean (SD)* | 74.0 (10.3) | | 70.2 (12.3) | | 0.416 |
| **Mother relationship status- T1** | *In relationship with father* | 294 | 100.0% | 159 | 94.6% | NA* |
|  | *Not in relationship with father* | 0 | 0.0% | 9 | 5.4% |  |
| **Mother's age at conception, years** | *Mean (SD)* | 32.7 (3.7) | | 32.1 (4.0) | | 0.135 |
| **Mother's ethnicity** | *White* | 289 | 97.0% | 156 | 94.5% | 0.295 |
|  | *Other* | 9 | 3.0% | 9 | 5.5% |  |
| **Mother's level of education** | *Baccalaureate +5 and more* | 181 | 60.5% | 91 | 49.7% | 0.039 |
|  | *Baccalaureate +3 to 4 years* | 75 | 25.1% | 52 | 28.4% |  |
|  | *Baccalaureate or lower* | 43 | 14.4% | 40 | 21.9% |  |
| **Mother employment status - T1** | *Employed* | 258 | 87.8% | 126 | 84.0% | 0.343 |
|  | *Unemployed* | 36 | 12.2% | 24 | 16.0% |  |
| **Mother employment status - 1 year** | *Employed* | 200 | 95.7% | 73 | 94.8% | NA* |
|  | *Unemployed* | 9 | 4.3% | 4 | 5.2% |  |
| **Mother professional skill class*** | *Skill level 3-4* | 264 | 90.7% | 122 | 81.9% | 0.012 |
|  | *Skill level 2* | 27 | 9.3% | 27 | 18.1% |  |
|  | *Skill level 1* | - | - | - | - |  |
|  | *Armed forces* | - | - | - | - |  |
| **Mother's weight (kg) - T1** | *Mean (SD)* | 63.7 (10.1) | | 65.5 (11.9) | | 0.066 |
| **Mother's tobacco consumption during pregnancy** | *1 or less cigarettes /day* | 273 | 96.8% | 135 | 92.5% | 0.076 |
|  | *Over 1 cigarette /day* | 9 | 3.2% | 11 | 7.5% |  |
| **Mother alcohol during pregnancy** | *No* | 91 | 30.4% | 58 | 35.8% | 0.366 |
|  | *One or less drinks per month* | 108 | 36.1% | 49 | 30.2% |  |
|  | *Over one drink per month* | 100 | 33.4% | 55 | 34.0% |  |
| **Mothers' prenatal mental health score (HADS, T3)** | *Mean (SD)* | 10.6 (4.9) | | 10.6 (5.2) | | 0.898 |
| **Mothers' postnatal mental health score (HADS, 1 year)** | *Mean (SD)* | 11.4 (4.9) | | 10.5 (4.9) | | 0.184 |
| **Diagnosed complications during pregnancy** | *No* | 266 | 89.6% | 155 | 86.6% | 0.404 |
|  | *Yes* | 31 | 10.4% | 24 | 13.4% |  |
| **Gestational duration (weeks)**** | *Mean (SD)* | 39.7 (1.4) | | 39.7 (1.8) | | 0.553 |
| **Birth weight (child), g** | *Mean (SD)* | 3278.2 (417.1) | | 3301.0 (512.2) | | 0.596 |
| **Delivery mode** | *Vaginal* | 259 | 86.0% | 139 | 78.1% | 0.034 |
|  | *Caesarean* | 42 | 14.0% | 39 | 21.9% |  |
| **Mother's parity at birth** | *0* | 142 | 47.2% | 80 | 43.7% | 0.759 |
|  | *1* | 130 | 43.2% | 84 | 45.9% |  |
|  | *2* | 29 | 9.6% | 19 | 10.4% |  |
| **No. of weeks breastfed before week 48** | *Mean (SD)* | 27.0 (15.9) | | 25.7 (15.9) | | 0.407 |
| **Number of hospitalisations between birth and 2 months old** | *0* | 273 | 91.9% | 142 | 88.2% | NA* |
|  | *1* | 23 | 7.7% | 16 | 9.9% |  |
|  | *2* | 1 | 0.3% | 3 | 1.9% |  |
| **Number of hospitalisations between 2 and 12 months old** | *0* | 282 | 96.9% | 130 | 97.7% | NA* |
|  | *1* | 9 | 3.1% | 2 | 1.5% |  |
|  | *2* | 0 | 0.0% | 1 | 0.8% |  |
| **Main mode of child care at 6 months** | *Collective day care* | 173 | 60.9% | 80 | 67.2% | 0.279 |
|  | *Other* | 111 | 39.1% | 39 | 32.8% |  |
| **Main mode of child care at 12 months** | *Collective day care* | 227 | 79.9% | 99 | 83.2% | 0.534 |
|  | *Other* | 57 | 20.1% | 20 | 16.8% |  |
| **Number of children in household (inc. part-time) – T1** | *Mean (SD)* | 0.7 (0.7) | | 0.8 (0.8) | | 0.155 |
| **Number of brothers and sisters - 1 year** | *Mean (SD)* | 0.7 (0.7) | | 0.8 (0.7) | | 0.391 |
| **Paternal mental health** | | | | | | |
|  |  | **n** | **%** | **Mean (SD)** | **n** | **p value** |
| **Prenatal Anxiety***** | *No* | 121 | 72.9% | - | - | - |
|  | *Yes* | 45 | 27.1% | - | - | - |
| **Prenatal Depression***** | *No* | 121 | 72.9% | - | - | - |
|  | *Yes* | 45 | 27.1% | - | - | - |
| **Postnatal Anxiety*** - 0-12 months** | *No* | 154 | 74.4% | - | - | - |
|  | *Yes* | 53 | 25.6% | - | - | - |
| **Postnatal Depression*** - 0-12 months** | *No* | 154 | 74.4% | - | - | - |
|  | *Yes* | 53 | 25.6% | - | - | - |
| **Postnatal Anxiety*** - 12-24 months** | *No* | 154 | 74.4% | - | - | - |
|  | *Yes* | 53 | 25.6% | - | - | - |
| **Postnatal Depression*** - 12-24 months** | *No* | 154 | 74.4% | - | - | - |
|  | *Yes* | 53 | 25.6% | - | - | - |
| **Child developmental outcomes** | | | | | | |
|  |  | **n** | **Mean (SD)** | **n** | **Mean (SD)** | **p value** |
| **Child Behaviour Checklist (CBCL)** | *Emotional reactivity* | 286 | 0.5 (0.3) | 131 | 0.5 (0.3) | 0.572 |
|  | *Anxiety/Depression* | 286 | 0.3 (0.3) | 131 | 0.3 (0.3) | 0.722 |
|  | *Somatic complaints* | 285 | 0.4 (0.3) | 130 | 0.4 (0.3) | 0.116 |
|  | *Withdrawn behaviour* | 286 | 0.2 (0.2) | 131 | 0.2 (0.2) | 0.178 |
|  | *Sleep problems* | 286 | 0.5 (0.3) | 131 | 0.5 (0.3) | 0.764 |
|  | *Attention problems* | 286 | 0.5 (0.2) | 129 | 0.5 (0.2) | 0.526 |
|  | *Aggressive behaviour* | 286 | 1.0 (0.2) | 130 | 1.0 (0.3) | 0.035 |
|  | *Other problems* | 286 | 0.9 (0.2) | 130 | 0.9 (0.3) | 0.088 |
|  | *Internalising problems* | 286 | 0.8 (0.3) | 131 | 0.8 (0.3) | 0.361 |
|  | *Externalising problems* | 286 | 1.1 (0.2) | 130 | 1.1 (0.3) | 0.048 |
|  | *Total score* | 286 | 32.5 (14.4) | 130 | 30.8 (16.0) | 0.284 |
| **Wechsler Primary and Preschool Scales of Intelligence (WPPSI)** | *Verbal comprehension index* | 265 | 112.2 (11.7) | 116 | 112.2 (11.4) | 0.990 |
|  | *Visuospatial index* | 266 | 110.4 (10.6) | 116 | 110.7 (11.1) | 0.796 |
|  | *Working memory index* | 264 | 105.7 (10.7) | 116 | 107.5 (10.8) | 0.132 |
|  | *Total Intelligence Quotient (IQ)* | 265 | 112.9 (11.0) | 116 | 113.6 (11.2) | 0.561 |
| **Behaviour Rating Inventory of Executive Function, Preschool version (BRIEF-P)** | *Inhibitory control and impulsivity* | 279 | 4.9 (0.5) | 126 | 4.8 (0.6) | 0.776 |
|  | *Shift* | 284 | 1.1 (0.1) | 130 | 1.1 (0.1) | 0.142 |
|  | *Emotional control* | 284 | 3.8 (0.5) | 130 | 3.8 (0.4) | 0.110 |
|  | *Working memory* | 277 | 1.3 (0.1) | 129 | 1.3 (0.1) | 0.306 |
|  | *Planning/organisation* | 282 | 3.7 (0.4) | 129 | 3.7 (0.4) | 0.932 |
| **Social Responsiveness Scale (SRS)** | *Social Awareness* | 280 | 5.8 (2.7) | 127 | 5.3 (2.8) | 0.095 |
|  | *Social Cognition* | 280 | 2.4 (0.7) | 127 | 2.3 (0.7) | 0.167 |
|  | *Social Communication* | 280 | 0.9 (0.2) | 127 | 0.9 (0.2) | 0.932 |
|  | *Social Motivation* | 280 | 0.8 (0.2) | 127 | 0.8 (0.3) | 0.460 |
|  | *Repetitive Behaviour (RRB)* | 280 | 0.6 (0.3) | 127 | 0.6 (0.3) | 0.300 |
|  | *SRS Total* | 280 | 1.4 (0.2) | 127 | 1.4 (0.2) | 0.349 |
| *Chi-square not calculated for variables containing groups where n<5. T1/T2/T3= First/Second/Third trimester. *Based on ISCO scoring. Skill level 1: Elementary occupations; Skill level 2: Clerical support workers, service and sales workers, agricultural, forestry and fishery workers, craft and related trade workers, plant and machine operators and assemblers; Skill levels 3-4: Managers, professionals, technicians and associate professionals. **Calculated from mother's last menstrual period ***Hospital Anxiety and Depression Scale (HADS); cut-off score of 8.* | | | | | | |
